# Characteristics of Escherichia coli Urine Isolates and Risk Factors for Secondary Bloodstream Infections in Patients with Urinary Tract Infections

**DOI:** 10.1128/spectrum.01660-22

**Published:** 2022-07-07

**Authors:** Hyeon Jin Choi, Seok Hoon Jeong, Kyeong Seob Shin, Young Ah Kim, Young Ree Kim, Hyun Soo Kim, Jong Hee Shin, Jeong Hwan Shin, Young Uh, Songmee Bae, Eun-Jeong Yoon, Jung Sik Yoo

**Affiliations:** a Korea National Institute of Health, Korea Disease Control and Prevention Agency, Cheongju-si, South Korea; b Department of Laboratory Medicine, Yonsei University College of Medicine, Seoul, South Korea; c Department of Laboratory Medicine, Chungbuk National University College of Medicine, Cheongju, South Korea; d Department of Laboratory Medicine, National Health Insurance Service, Ilsan Hospital, Goyang, South Korea; e Department of Laboratory Medicine, Jeju National University College of Medicine, Jeju, South Korea; f Department of Laboratory Medicine, Hallym University Dongtan Sacred Heart Hospital, Hallym University College of Medicine, Hwaseong, South Korea; g Department of Laboratory Medicine, Chonnam National University Medical School, Gwangju, South Korea; h Department of Laboratory Medicine, Inje University College of Medicine, Busan, South Korea; i Department of Laboratory Medicine, Yonsei University Wonju College of Medicine, Wonju, South Korea; j Research Institute of Bacterial Resistance, Yonsei University College of Medicine, Seoul, South Korea; k Paik Institute for Clinical Research, Inje University College of Medicine, Busan, South Korea; Johns Hopkins Hospital

**Keywords:** *Escherichia coli*, urinary tract infections, ST131 H41 sublineage, CTX-M ESBL, combination therapy

## Abstract

Escherichia coli is responsible for more than 80% of all incidences of urinary tract infections (UTIs). We assessed a total of 636 cases of patients with E. coli UTIs occurring in June 2019 in eight tertiary hospitals in South Korea for the traits of patients with E. coli UTIs, UTI-causative E. coli isolates, and risk factors associated with bloodstream infections (BSIs) secondary to UTIs. Antimicrobial susceptibility testing was conducted using the disc diffusion method, and the genes for extended-spectrum beta-lactamases (ESBLs) and plasmid-mediated *ampC* genes were screened by using PCR and sequencing. Multilocus sequence typing and virulence pheno-/genotyping were carried out. A total of 49 cases developed BSIs. The E. coli urine isolates primarily comprised sequence type 131 (ST131) (30.0%), followed by ST1193, ST95, ST73, and ST69. Three-quarters of the ST131 H30Rx isolates possessed the *bla*_CTX-M-15_-like gene, whereas 66% of H30R and 50% of H41 isolates possessed the *bla*_CTX-M-14_-like gene. All the ST1193 isolates showed biofilm formation ability, and three-quarters of the ST73 isolates exhibited hemolytic activity with high proportions of *papC*, *focG*, and *cnf1* positivity. The prevalence of the ST131 H41 sublineage and its abundant CTX-M possession among the E. coli urine isolates were noteworthy; however, no specific STs were associated with bloodstream invasion. For BSIs secondary to UTIs, the *papC* gene was likely identified as a UTI-causative E. coli-related risk factor and urogenital cancer (odds ratio [OR], 12.328), indwelling catheter (OR, 3.218), and costovertebral angle tenderness (OR, 2.779) were patient-related risk factors.

**IMPORTANCE** Approximately half of the BSIs caused by E. coli are secondary to E. coli UTIs. Since the uropathogenic E. coli causing most of the UTIs is genetically diverse, understanding the risk factors in the E. coli urine isolates causing the BSI is important for pathophysiology. Although the UTIs are some of the most common bacterial infectious diseases, and the BSIs secondary to the UTIs are commonly caused by E. coli, the assessments to find the risk factors are mostly focused on the condition of patients, not on the bacterial pathogens. Molecular epidemiology of the UTI-causative E. coli pathogens, together with the characterization of the E. coli urine isolates associated with the BSI secondary to UTI, was carried out, suggesting treatment options for the prevalent antimicrobial-resistant organisms.

## INTRODUCTION

Urinary tract infections (UTIs) are among the most common bacterial infections, annually affecting 150 million patients globally (1). UTIs are caused by both Gram-negative and Gram-positive bacteria, as well as by certain fungi. Among these, the most common UTI-causing agent is uropathogenic Escherichia coli (UPEC), accounting for approximately 80% of UTI cases (2). UPEC isolates are often clonal with the globally prevalent sequence types (STs), including ST131, ST69, ST73, and ST95 (3). The pathogenesis of UTIs by UPEC involves (i) bacterial colonization of the periurethral area and the urethra, (ii) ascending bacterial infection of the bladder, (iii) bacterial adhesion to the surface and interaction with the bladder, (iv) bacterial invasion and replication by formation of an intracellular biofilm and residence in the underlying urothelium, and (v) kidney colonization and host tissue damage with increased risk of a bloodstream infection (BSI) secondary to the UTI (1). The secondary BSIs, which are defined as an infection developed from a detectable infection as a source of the bacteremia, are linked with consequences of high morbidity/mortality, lengthened hospital stay, and associated costs (4).

Known bacterial pathogen-related risk factors involved in BSIs secondary to the UTI are adhesins, toxins, surface polysaccharides, flagella, and iron acquisition systems, which are associated with any of the pathogenesis steps (2, 5). Therefore, many efforts are made to further develop targeted antivirulence drugs and to devise effective strategies, such as combination therapy, to treat the infections (6). Antimicrobial resistance, which often leads to a treatment failure through limited therapeutic options for the patients with UTIs, is another risk factor for secondary BSIs (7). The patient-related risk factors, which were assessed for unrestricted infection-causative bacterial species, were age, male sex, indwelling urethral catheter, underlying diseases such as diabetes mellitus and malignancy, and the length of hospitalizations (8–11).

The aim of the study was to investigate the clonal diversity, antimicrobial resistance, virulence pheno- and genotypes of E. coli urine isolates, and the pathogen- and patient-associated factors leading to BSIs secondary to UTI by analyzing E. coli clinical isolates retrieved from deduplicated UTI cases which occurred in eight tertiary care hospitals in a month. In addition, possible treatment options for UTIs caused by CTX-M-type extended-spectrum beta-lactamase (ESBL)-producing E. coli were proposed through *in vitro* synergy testing of cephalosporin and aminoglycoside drugs.

## RESULTS

### Characteristics of enrolled patients with UTIs.

A total of 636 E. coli UTI cases occurring in a month was gathered from eight sentinel hospitals. The median value of the E. coli UTI cases per hospital was 88.5 (30 to 121 by hospital). Of the total patients with E. coli UTI, 72.7% (464/636) were female and 48.4% (308/636) were elderly patients (>65 years). Over four-fifths of the UTIs (85.7%, 547/636) were infections of community origin (CO) (Table 1). Mortality of the patient was observed in 4 cases by 30 days and 6 cases by 90 days after the UTI onset; moreover, 49 of the 636 UTI cases (7.7%) developed secondary BSIs, with 5 to 12 cases per hospital.

**TABLE 1:** Characteristics of the enrolled patients with Escherichia coli UTI^*a*^

Characteristic	Total (*n* = 636)		sBSI^*b*^ (*n* = 49)		Other^*c*^ (*n* = 587)		OR	95% CI	*P* value
	No.	%	No.	%	No.	%			
Demography									
**Inpatients**	**313**	**49.2**	**37**	**75.5**	**276**	**47.0**	**3.468**	**1.727–7.456**	**<0.001**
CO	545	85.7	40	81.6	420	71.6	0.709	0.320–1.740	0.390
Female	462	72.6	36	73.5	426	72.6	1.047	0.526–2.208	1.000
**Old age (>65 yr)**	**308**	**48.4**	**41**	**83.7**	**267**	**45.5**	**6.126**	**2.773–15.397**	**<0.001**
90-day mortality	6	0.9	5	10.2	1	0.2	2.420	0.050–22.263	0.383
Underlying diseases									
Diabetes mellitus	112	17.6	12	24.5	100	17.0	1.578	0.723–3.228	0.239
Chronic renal diseases	52	8.2	4	8.2	48	8.2	0.998	0.250–2.916	1.000
Other cancer	40	6.3	3	6.1	37	6.3	0.969	0.184–3.251	1.000
**Urogenital cancer**	**4**	**0.6**	**2**	**4.1**	**2**	**0.3**	**12.328**	**0.876–173.481**	**0.032**
Immunosuppression treatment	25	3.9	3	6.1	22	3.7	1.673	0.309–5.889	0.431
Symptom									
**Indwelling catheter**	**100**	**15.7**	**17**	**36.7**	**83**	**14.1**	**3.218**	**1.600–6.288**	**0.001**
**Dysuria**	**89**	**14.0**	**1**	**2.0**	**88**	**15.0**	**0.118**	**0.003–0.711**	**0.009**
Frequency	72	11.3	5	10.2	67	11.4	0.882	0.264–2.330	1.000
Urgency	33	5.2	1	2.0	32	5.5	0.362	0.009–2.269	0.503
Flank pain	39	6.1	4	8.2	35	6.0	1.401	0.346–4.181	0.531
**Fever**	**116**	**26.1**	**38**	**77.6**	**128**	**21.8**	**12.329**	**5.967–27.524**	**<0.001**
**CVA tenderness**	**34**	**5.3**	**6**	**12.2**	**28**	**4.8**	**2.779**	**0.892–7.351**	**0.039**
Nausea	20	3.1	1	2.0	19	3.2	0.623	0.015–4.094	1.000
Vomiting	28	4.4	3	6.1	25	4.3	1.465	0.273–5.083	0.469
Lab data^*d*^									
**WBC**	**280**	**44.0**	**34**	**69.4**	**295**	**50.3**	**3.136**	**1.621–6.341**	**<0.001**
**Hemoglobin**	**335**	**52.7**	**40**	**81.6**	**295**	**50.3**	**4.391**	**2.050–10.482**	**<0.001**
**Platelets**	**146**	**23.0**	**19**	**38.8**	**127**	**21.6**	**2.29**	**1.177–4.366**	**0.012**
**Bilirubin**	**105**	**16.5**	**19**	**38.8**	**86**	**14.7**	**3.679**	**1.868–7.106**	**<0.001**
CRP	29	4.6	4	8.2	25	4.3	1.995	0.484–6.147	0.270
Status									
**Abnormal mental status**	**23**	**3.6**	**7**	**14.3**	**16**	**2.7**	**5.917**	**1.948–16.253**	**<0.001**
**Mechanical ventilation**	**27**	**4.2**	**7**	**14.3**	**20**	**3.4**	**4.705**	**1.589–12.435**	**0.003**
Empirical treatment									
ES cephalosporins	277	43.6	23	46.9	254	43.3	1.159	0.616–2.169	0.654
Carbapenems	36	5.7	6	12.2	30	5.1	2.585	0.834–6.788	0.050
Fluoroquinolones	113	17.8	11	22.4	102	17.4	1.376	0.613–2.862	0.435
Aminoglycosides	14	2.2	1	2.0	13	2.2	0.92	0.021–6.378	1.000
Definitive treatment									
ES cephalosporins	189	29.7	15	30.6	174	29.6	1.047	0.516–2.035	0.872
**Carbapenems**	**82**	**12.9**	**16**	**32.7**	**66**	**11.2**	**3.816**	**1.856–7.593**	**<0.001**
Fluoroquinolones	95	14.9	12	24.5	83	14.1	1.967	0.896–4.052	0.060
Aminoglycosides	13	2.0	3	6.1	10	1.7	3.750	0.641–15.256	0.071
Data for the causative E. coli isolate									
MLST									
ST131	191	30.0	21	42.9	170	29.0	1.838	0.963–3.460	0.051
ST131 H41	50	7.9	5	10.2	45	7.7	1.368	0.403–3.691	0.576
ST131 H30R	78	12.3	10	20.4	68	11.6	1.955	0.831–4.219	0.108
ST131 H30Rx	56	8.8	4	8.2	52	8.9	0.915	0.230–2.660	1.000
ST1193	90	14.2	5	10.2	85	14.5	0.671	0.202–1.758	0.525
ST95	63	9.9	6	12.2	57	9.7	1.297	0.432–3.246	0.616
ST73	55	8.6	5	10.2	50	8.5	1.220	0.361–3.271	0.602
ST69	50	7.9	7	14.3	43	7.3	2.105	0.753–5.119	0.094
Resistance type									
MDR	138	21.7	6	12.2	132	22.5	0.481	0.164–1.170	0.106
XDR	291	45.8	26	53.1	265	45.1	1.373	0.734–2.583	0.300
Resistance to drugs									
ES cephalosporins	239	37.6	23	46.9	216	36.8	1.518	0.806–2.845	0.169
Fluoroquinolones	294	46.2	25	51.0	269	45.8	1.231	0.658–2.309	0.552
Aminoglycosides	192	30.2	16	32.7	176	30.0	1.132	0.566–2.180	0.746
Beta-lactamases									
Plasmid-mediated AmpC	9	1.4	1	2.0	8	1.4	1.507	0.033–11.641	0.516
All ESBLs	220	34.6	22	44.9	198	33.7	1.600	0.845–3.001	0.120
Group 1 CTX-M	84	13.2	10	20.4	74	12.6	1.776	0.758–3.818	0.126
Group 9 CTX-M	141	22.2	12	24.5	129	22.0	1.151	0.531–2.337	0.720
Virulence phenotype									
Hemolysis	168	26.4	14	28.6	154	26.2	1.124	0.544–2.213	0.737
Biofilm formation	459	72.2	34	69.4	425	72.4	0.864	0.444–1.756	0.623
Virulence genotype									
*afaA*	72	11.3	8	16.3	64	10.9	1.593	0.617–3.644	0.243
*cnf1*	166	26.1	15	30.6	151	25.7	1.273	0.626–2.481	0.498
*hlyF*	38	6.0	1	2.0	37	6.3	0.310	0.007–1.927	0.349
*sat*	292	45.9	28	57.1	264	45.0	1.630	0.870–3.095	0.104
***papC***	**259**	**40.7**	**27**	**55.1**	**232**	**39.5**	**1.876**	**1.002–3.547**	**0.035**
*focG*	56	8.8	4	8.2	52	8.9	0.915	0.230–2.660	1.000

a The odds ratio, the 95% CI, and the *P* values were estimated by Fisher’s exact tests. The factors with statistical significance (*P* < 0.05) are indicated in boldface. CO, community-originating infections; CVA, costovertebral angle; WBC, white blood cells; CRP, C-reactive protein; ES, extended spectrum; CI, confidence interval; OR, odds ratio.

b sBSI, the BSI cases secondary to UTIs.

c Other than the BSI cases secondary to UTIs.

d The cases with values out of the normal range of each type of data were taken into account.

Diabetes mellitus was dominant (17.6%, 112/636) among underlying diseases of the patients with UTIs, followed by chronic renal diseases (8.2%, 52/636) (Table 1). Urogenital cancer, i.e., kidney and/or bladder cancer, was a risk factor for the BSI secondary to UTI with an odds ratio (OR) of 12.328 (95% confidence interval [CI], 0.876 to 173.481; *P* = 0.032). UTI patients with an indwelling catheter (OR, 3.218; 95% CI, 1.600 to 6.288; *P* = 0.001) and costovertebral angle (CVA) tenderness (OR, 2.779; 95% CI, 0.892 to 7.351; *P* = 0.039) were patient-associated risk factors with the BSIs secondary to UTI, whereas dysuria (OR, 0.118; 95% CI, 0.003 to 0.711; *P* = 0.009) was instead a protective factor. Among the lab data, numbers of white blood cells (WBC) and platelets and the levels of hemoglobin and bilirubin, which were out of normal ranges, were possible signs for the BSIs secondary to UTI.

### Strain types of E. coli urine isolates.

Among 83 different STs identified in the study, ST131 (30.0%, 191/636) was the primary dominant ST, followed by ST1193 (14.1%, 90/636), ST95 (9.9%, 63/636), ST73 (8.6%, 55/636), ST69 (7.9%, 50/636), and other STs consisting of fewer than 20 isolates. Of a total of 191 ST131 isolates of clonal complex (CC) 131, 29.3% (*n* = 56), 40.8% (*n* = 78), and 26.2% (*n* = 50) were H30Rx, H30R, and H41 subgroups, respectively, whereas the other subgroups including H30 (*n* = 2), H22 (*n* = 2), H38 (*n* = 1), H43 (*n* = 1), and H47 (*n* = 1) were found rarely.

**FIG 1:**
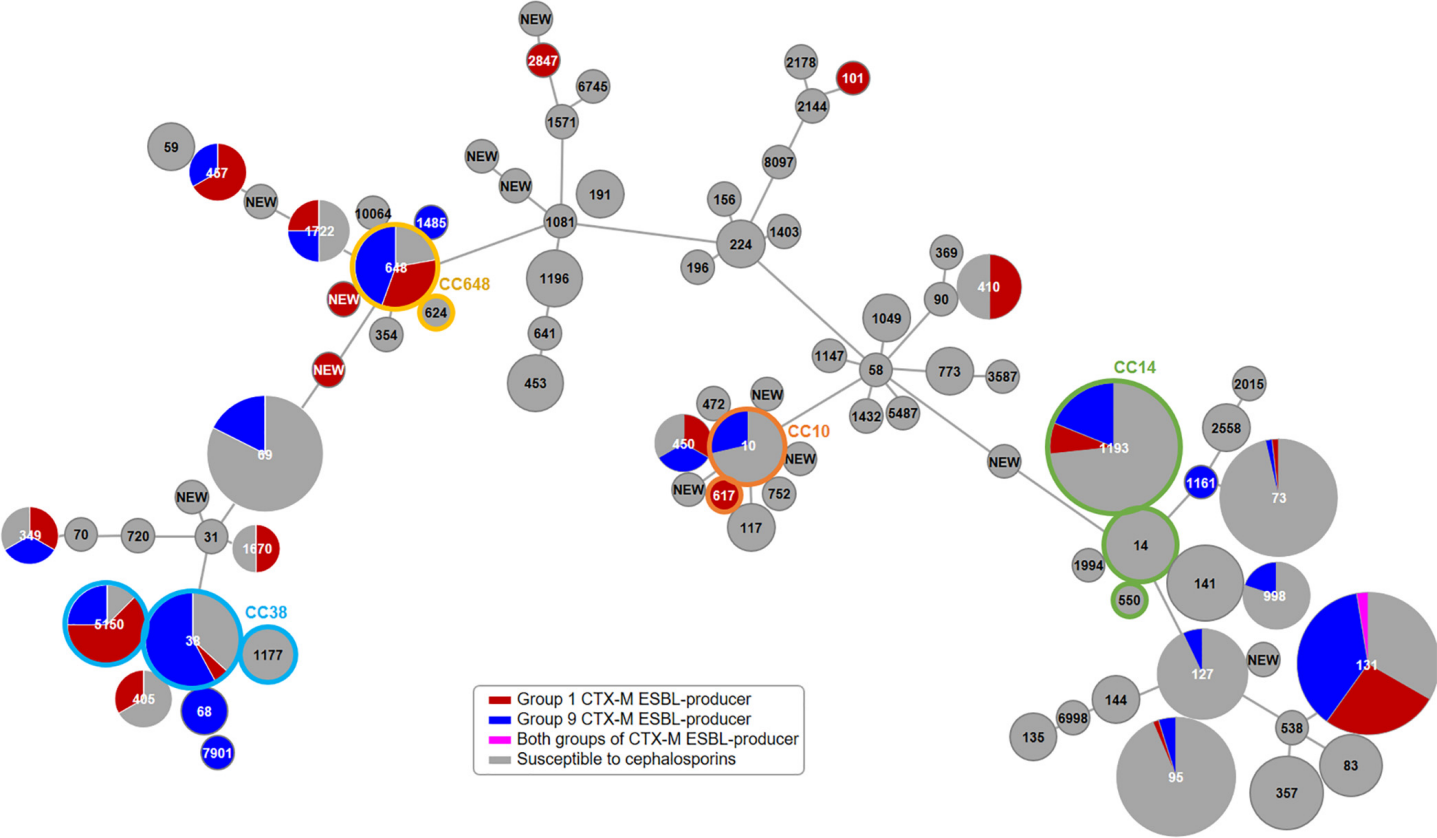
Minimum spanning tree of the Escherichia coli urine isolates. The number in the center of each circle indicates the ST, the size of each circle indicates the logarithmic number of isolates belonging to the ST, and the pie graph indicates the proportion of the *bla*_CTX-M-15_-like and the *bla*_CTX-M-14_-like genes possessed by the ST isolates. Clonal groups are indicated with colored outlines of each circle if available. The length of the branch represents the number of allele differences between STs.

CC14 (14.9%, 98/636), which was composed of ST1193 (*n* = 90), ST14 (*n* = 7), and ST550 (*n* = 1), was the second most dominant CC, and CC38 (of ST38, ST5150, and ST1177; *n* = 20), CC648 (of ST648 and ST624; *n* = 10), and CC10 (of ST10 and ST617; *n* = 8) were next (Fig. 1).

### Antimicrobial resistance characteristics of E. coli urine isolates.

Among the E. coli urine isolates, 21.7% (138/636) were multidrug resistant (MDR) and 45.8% (291/636) were extensively drug resistant (XDR). Ampicillin and piperacillin resistance was observed in 73.4% (467/636) and 68.7% (437/636) of the E. coli isolates, respectively. Rates of resistance to cefotaxime, ceftazidime, and cefepime were 39.2% (249/636), 16.7% (106/636), and 34.7% (221/636), respectively, and that to cefoxitin was 8.5% (54/636), whereas only one isolate was resistant to carbapenems. Ciprofloxacin resistance was observed in 46.2% (294/636) of the isolates. Gentamicin and amikacin resistance was observed in 29.9% (190/636) and 1.6% (10/636) of the isolates, respectively. One isolate was resistant to tigecycline, and none were resistant to colistin.

Among the 249 cefotaxime-resistant isolates, 220 carried the *bla*_CTX-M_ gene: 79 isolates carried the *bla*_CTX-M-15_-like gene, 136 isolates carried the *bla*_CTX-M-14_-like gene, and five isolates carried both genes. Among the 54 cefoxitin-resistant isolates, 9 isolates possessed a gene for plasmid-mediated AmpC, either the *bla*_DHA_ (*n* = 4) or the *bla*_CMY-2_ (*n* = 5) gene.

### Virulence phenotypic and genotypic characteristics of E. coli urine isolates.

Hemolytic activities were observed in 26.4% (168/636) of the E. coli urine isolates, and the biofilm formation ability was found in 72.2% (459/636) of the isolates. Among the four adhesion genes, the *papC* gene was the most abundant, identified in 40.7% (259/636) of the E. coli isolates, followed by *afa* (11.3%, 72/636) and *focG* (8.8%, 56/636). Among the toxin genes, the *sat* gene was the most abundant, identified in 45.9% (292/636) of the E. coli urine isolates, followed by *cnf1* (26.1%, 166/636) and *hlyF* (6.0%, 38/636).

### Clonal traits.

Clonal traits of the five dominant STs are presented in Table 2. The prevalent ST131 presented higher-than-average rates of resistance to all tested drugs, except cefoxitin. Approximately three-quarters of the ST131 isolates were XDR (74.9%, 143/192); the sublineages H30Rx (91.1%, 51/56) and H30R (80.8%, 63/78) had a higher proportion of XDR isolates than that in the remaining lineages. ST131 H41 presented high rates of XDR and MDR at 54.0% and 30.0% of the isolates, respectively. Of the ST131 isolates, 27.7% (*n* = 53) and 41.9% (*n* = 80) possessed the *bla*_CTX-M-15_-like and the *bla*_CTX-M-14_-like genes, respectively. The sublineage H30Rx isolates more frequently carried the *bla*_CTX-M-15_-like gene than the *bla*_CTX-M-14_-like gene (78.6% versus 16.1%), while the sublineage H30R less frequently carried the *bla*_CTX-M-15_-like gene than the *bla*_CTX-M-14_-like gene (9.0% versus 60.3%). As a notable clonal feature in the urine isolates, half of the sublineage H41 isolates possessed the *bla*_CTX-M-14_-like gene (48.0%, 24/50). All the plasmid-mediated *ampC* genes were identified in ST131 isolates. Of the 191 ST131 isolates, all but two had biofilm-forming ability (99.0%, 189/191) and double the proportion of the sublineage H30Rx isolates had hemolysis activity (53.6%, 30/56) compared to that of the other ST131 sublineages. The virulence-associated genes, except *focG* and *hlyF*, were more frequently identified in ST131 clones.

**TABLE 2:** Characteristics of the Escherichia coli urine isolates belonging to the dominant STs^*a*^

Characteristic	Total (*n* = 636)		ST131 (*n* = 191)			ST131 H30Rx (*n* = 56)			ST131 H30R (*n* = 78)			ST131 H41 (*n* = 50)			ST1193 (*n* = 90)			ST95 (*n* = 63)			ST73 (*n* = 55)			ST69 (*n* = 50)		
	No.	%	No.	%	*P* value	No.	%	*P* value	No.	%	*P* value	No.	%	*P* value	No.	%	*P* value	No.	%	*P* value	No.	%	*P* value	No.	%	*P* value
Demography																										
Female	462	72.6	135	70.7	0.529	37	66.1	0.318	58	74.4	0.820	35	70.0	0.786	68	75.6	0.588	44	69.8	0.707	36	65.5	0.275	38	76.0	0.697
Old age (>65 yr)	308	48.4	**108**	**56.5**	**0.009**	**37**	**66.1**	**0.009**	46	59.0	0.062	20	40.0	0.274	43	47.8	0.985	**22**	**34.9**	**0.033**	30	54.5	0.419	21	42.0	0.424
CO infections	545	85.7	157	82.2	0.127	44	78.6	0.163	64	82.1	0.419	42	84.0	0.884	78	86.7	0.902	**60**	**95.2**	**0.037**	50	90.9	0.340	42	84.0	0.884
Inpatients	313	49.2	94	49.2	1.000	30	53.6	0.587	40	51.3	0.788	20	40.0	0.226	39	43.3	0.276	28	44.4	0.506	26	47.3	0.873	30	60.0	0.149
Secondary BSI	49	7.7	21	11.0	0.061	4	7.1	1.000	10	12.8	0.114	5	10.0	0.720	5	5.6	0.541	6	9.5	0.748	5	9.1	0.890	7	14.0	0.144
Resistance to drug																										
Ampicillin	467	73.4	**181**	**94.8**	**<0.001**	**54**	**96.4**	**<0.001**	**74**	**94.9**	**<0.001**	**48**	**96.0**	**<0.001**	73	81.1	0.098	**25**	**39.7**	**<0.001**	**32**	**58.2**	**0.012**	**44**	**88.0**	**0.024**
Piperacillin	437	68.7	**173**	**90.6**	**<0.001**	**52**	**92.9**	**<0.001**	**71**	**91.0**	**<0.001**	**47**	**94.0**	**<0.001**	**71**	**78.9**	**0.034**	**20**	**31.7**	**<0.001**	**29**	**52.7**	**0.012**	**42**	**84.0**	**0.023**
Ceftizoxime	422	66.4	**177**	**92.7**	**<0.001**	**55**	**98.2**	**<0.001**	**72**	**92.3**	**<0.001**	**46**	**92.0**	**<0.001**	54	60.0	0.209	**17**	**27.0**	**<0.001**	**23**	**41.8**	**<0.001**	**41**	**82.0**	**0.022**
Cefotaxime	249	39.2	**136**	**71.2**	**<0.001**	**49**	**87.5**	**<0.001**	**55**	**70.5**	**<0.001**	**30**	**60.0**	**0.003**	**25**	**27.8**	**0.023**	**4**	**6.3**	**<0.001**	**3**	**5.5**	**<0.001**	15	30.0	0.219
Ceftazidime	106	16.7	**60**	**31.4**	**<0.001**	**40**	**71.4**	**<0.001**	12	15.4	0.871	6	12.0	0.469	**5**	**5.6**	**0.004**	**1**	**1.6**	**0.001**	**2**	**3.6**	**0.012**	**2**	**4.0**	**0.021**
Cefepime	221	34.7	**129**	**67.5**	**<0.001**	**48**	**85.7**	**<0.001**	**54**	**69.2**	**<0.001**	**27**	**54.0**	**0.005**	23	25.6	0.063	**4**	**6.3**	**<0.001**	**2**	**3.6**	**<0.001**	**8**	**16.0**	**0.006**
Aztreonam	147	23.1	**97**	**50.8**	**<0.001**	**46**	**82.1**	**<0.001**	**29**	**37.2**	**0.003**	**21**	**42.0**	**0.002**	**8**	**8.9**	**0.001**	**1**	**1.6**	**<0.001**	**2**	**3.6**	**0.001**	**2**	**4.0**	**0.002**
Cefoxitin	54	8.5	13	6.8	0.399	5	8.9	1.000	3	3.8	0.176	3	6.0	0.694	3	3.3	0.091	4	6.3	0.686	1	1.8	0.109	6	12.0	0.507
Amikacin	10	1.6	**8**	**4.2**	**0.002**	**6**	**10.7**	**<0.001**	2	2.6	0.790	0	0	0.735	1	1.1	1.000	0	0	0.601	0	0	0.679	0	0	0.735
Gentamicin	190	29.9	**83**	**43.5**	**<0.001**	**31**	**55.4**	**<0.001**	31	39.7	0.057	21	42.0	0.073	35	38.9	0.058	**8**	**12.7**	**0.003**	11	20.0	0.129	20	40.0	0.142
Ciprofloxacin	294	46.2	**139**	**72.8**	**<0.001**	**56**	**100**	**<0.001**	**78**	**100**	**<0.001**	**5**	**10.0**	**<0.001**	**89**	**98.9**	**<0.001**	**0**	0	**<0.001**	**1**	**1.8**	**<0.001**	**9**	**18.0**	**<0.001**
Co-trimoxazole	266	41.8	**96**	**50.3**	**0.006**	22	39.3	0.794	**44**	**56.4**	**0.008**	**28**	**56.0**	**0.049**	45	50	0.114	**15**	**23.8**	**0.004**	11	20	0.001	**32**	**64.0**	**0.002**
Tigecycline	1	0.2	1	0.5	0.663	0	0	1.000	0	0	1.000	1	2.0	0.117	0	0	1.000	0	0	1.000	0	0	1.000	0	0	1.000
MDR	138	21.7	**31**	**16.2**	**0.037**	**4**	**7.1**	**0.009**	10	12.8	0.060	15	30.0	0.192	**30**	**33.3**	**0.006**	18	28.6	0.217	14	25.5	0.592	**18**	**36.0**	**0.017**
XDR	291	45.8	**143**	**74.9**	**<0.001**	**51**	**91.1**	**<0.001**	**63**	**80.8**	**<0.001**	27	54.0	0.284	47	52.2	0.224	**3**	**4.8**	**<0.001**	**10**	**18.2**	**<0.001**	18	36.0	0.196
Plasmidic AmpC	9	1.4	**9**	**4.7**	**<0.001**	**3**	**5.4**	**0.043**	1	1.3	1.000	**3**	**6.0**	**0.025**	0	0	1.000	0	0	0.660	0	0	0.740	0	0	0.796
CTX-M ESBL	220	34.6	**128**	**67**	**<0.001**	**48**	**85.7**	**<0.001**	**54**	**69.2**	**<0.001**	**26**	**52.0**	**0.011**	24	26.7	0.113	**4**	**6.3**	**<0.001**	**2**	**3.6**	**<0.001**	**9**	**18.0**	**0.016**
CTX-M-15-like	84	13.2	**53**	**27.7**	**<0.001**	**44**	**78.6**	**<0.001**	7	9.0	0.317	2	4.0	0.074	6	6.7	0.070	**1**	**1.6**	**0.007**	**1**	**1.8**	**0.016**	**0**	0	**0.008**
CTX-M-14-like	141	22.2	**80**	**41.9**	**<0.001**	9	16.1	0.326	**47**	**60.3**	**<0.001**	**24**	**48.0**	**<0.001**	18	20.0	0.691	**3**	**4.8**	**0.001**	**1**	**1.8**	**<0.001**	9	18.0	0.574
Virulence																										
Hemolysis	168	26.4	**65**	**34**	**0.006**	**30**	**53.6**	**<0.001**	21	26.9	1.000	14	28.0	0.922	**0**	0	**<0.001**	17	27	1.000	**42**	**76.4**	**<0.001**	**4**	**8.0**	**0.004**
Biofilm	459	72.2	**189**	**99**	**<0.001**	**55**	**98.2**	**<0.001**	**77**	**98.7**	**<0.001**	**50**	**100.0**	**<0.001**	**90**	**100**	**<0.001**	**1**	**1.6**	**<0.001**	**5**	**9.1**	**<0.001**	**44**	**88.0**	**0.015**
*afaA*	72	11.3	**38**	**19.9**	**<0.001**	7	12.5	0.944	**17**	**21.8**	**0.003**	**11**	**22.0**	**0.024**	**3**	**3.3**	**0.016**	**0**	0	**0.005**	**1**	**1.8**	**0.035**	2	4.0	0.142
*cnf1*	166	26.1	**73**	**38.2**	**<0.001**	**35**	**62.5**	**<0.001**	22	28.2	0.753	16	32.0	0.411	**0**	0	**<0.001**	16	25.4	1.000	**44**	**80**	**<0.001**	**0**	0	**<0.001**
*hlyF*	38	6.0	**0**	0	**<0.001**	0	0	0.093	**0**	0	**0.034**	0	0	0.122	1	1.1	0.063	**15**	**23.8**	**<0.001**	0	0	0.097	3	6.0	1.000
*sat*	292	45.9	**133**	**69.6**	**<0.001**	**43**	**76.8**	**<0.001**	**61**	**78.2**	**<0.001**	26	52.0	0.452	**78**	**86.7**	**<0.001**	**5**	**7.9**	**<0.001**	24	43.6	0.832	26	52.0	0.452
*papC*	259	40.7	**92**	**48.2**	**0.016**	**46**	**82.1**	**<0.001**	27	34.6	0.294	18	36.0	0.577	**2**	**2.2**	**<0.001**	**53**	**84.1**	**<0.001**	**42**	**76.4**	**<0.001**	24	48.0	0.347
*focG*	56	8.8	**1**	**0.5**	**<0.001**	**0**	0	**0.029**	**0**	0	**0.007**	**0**	0	**0.042**	**0**	0	**0.003**	1	1.6	0.058	**46**	**83.6**	**<0.001**	**0**	0	**0.042**

a The *P* values were estimated through Yates continuity-corrected Pearson’s chi-square test. The factors with statistical significance (*P* < 0.05) are indicated in boldface. Other subtypes include H22 (*n* = 2), H38 (*n* = 1), H43 (*n* = 1), and H47 (*n* = 1). Five of the isolates possessed both the group 1 and group 9 *bla*_CTX-M_ genes. One E. coli ST131 H43 isolate possessed the *bla*_DHA_ gene. One E. coli ST131 H48 isolate possessed the *bla*_CMY-2_ gene.

The second dominant clone, ST1193, presented a more proportional composition of MDR (33.3%, 30/90) and XDR (52.2%, 47/90) phenotypes. None of the ST1193 isolates presented hemolytic activity, whereas all were able to form biofilms. Fewer isolates carried virulence genes except *sat* (86.7%, 78/90).

The ST95 isolates were mostly identified from the CO infections (95.2%, 60/63), and few isolates presented XDR phenotypes (4.8%, 3/63). Only one isolate belonging to ST95 had the ability to form biofilms, and the highest proportion was observed for the *papC*- (84.1%, 53/63) and *hlyF*-positive (23.8%, 15/63) isolates.

Of the fourth most identified ST73 isolates, more than three-quarters of the isolates presented hemolytic activity (76.4%, 42/55), whereas 9.1% (5/55) of the isolates had biofilm-forming ability. More than three-quarters of the isolates had either the *papC* (76.4%, 42/55), *focG* (83.6%, 46/55), or *cnf1* (80.0%, 44/55) gene.

Among the ST69 isolates, fewer isolates had hemolytic activity (8.0%, 4/50) and more isolates had biofilm-forming ability (88.0%, 44/50).

### *In vitro* synergistic activity of the extended-spectrum cephalosporin-amikacin combination.

As the rates of resistance to extended-spectrum cephalosporins and amikacin were low in any E. coli urine clone, the efficacy of the drugs was evaluated alone or in combination. Similarly, the predominant ST131 clones having the dominant resistance determinants CTX-M-type ESBLs were taken into account for the *in vitro* synergistic activity.

Three ST131 isolates carrying three different types of the *bla*_CTX-M_ ESBL genes (D546-14 for the D0019EC0546 isolate carrying the *bla*_CTX-M-14_ gene, F434-15 for the F0019EC0434 isolate carrying the *bla*_CTX-M-15_ gene, and E777-27 for the E0019EC0777 isolate carrying the *bla*_CTX-M-27_ gene) were used to evaluate the efficacy of antimicrobial combinations for the three extended-spectrum cephalosporins—cefotaxime, ceftazidime, and cefepime with amikacin (Table 3). The MICs in D546-14, E777-27, and F434-15 for the drugs were as follows: cefotaxime, 32, 256, and 1,024 mg/L; ceftazidime, 1, 8, and 64 mg/L; and cefepime, 4, 8, and 128 mg/mL, respectively. All the isolates were susceptible to amikacin as the MICs were 4 or 8 mg/L. *In vitro* testing of the cefotaxime-amikacin combination indicated a synergistic activity against E777-27 and F434-15 isolates, with fractional inhibitory concentration (FIC) values of 0.3, whereas an additive activity was observed for the combination against D546-14 with the FIC value of 0.5. The ceftazidime-amikacin or cefepime-amikacin combination presented an additive effect against all three isolates.

**TABLE 3:** MICs and FIC indexes of cephalosporins with amikacin

Identifier	Isolate	CTX-M ESBL gene	MIC (μg/ml) (FIC index with amikacin)^*a*^			
			Cefotaxime	Ceftazidime	Cefepime	Amikacin
D546-14	D0019EC0546	*bla*_CTX-M-14_	32 (0.5)	1 (2.3)	4 (1)	4
E777-27	E0019EC0777	*bla*_CTX-M-27_	256 **(0.3)**	8 (0.6)	8 (0.6)	8
F434-15	F0019EC0434	*bla*_CTX-M-15_	1,024 **(0.3)**	64 (0.6)	128 (0.6)	8

a FIC indexes indicating synergistic effect are indicated in bold face.

**FIG 2:**
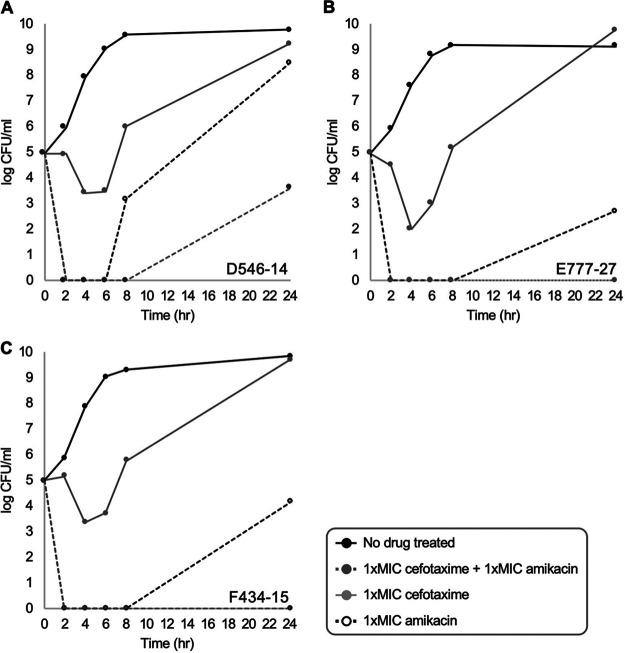
Time-kill kinetic assay of cefotaxime in combination with amikacin for the Escherichia coli ST131 isolates D546-14 carrying the *bla*_CTX-M-14_ gene (A), E777-27 carrying the *bla*_CTX-M-15_ gene (B), and F434-15 harboring the *bla*_CTX-M-27_ gene (C). Black circles with black lines indicate the bacterial growth in media devoid of any adjuvants, gray circles with solid lines and white circles with broken lines indicate the bacterial growth in media supplemented either with 1× MIC of cefotaxime or with 1× MIC of amikacin, respectively, and gray circles with broken lines present the bacterial growth in media supplemented with 1× MIC of cefotaxime and 1× MIC of amikacin.

Furthermore, a time-kill kinetic assay was carried out for the three isolates to better evaluate the cefotaxime and amikacin pharmacodynamic interaction (Fig. 2). The diminishing or increasing bacterial counts of the D546-14, E777-27, and F434-15 isolates over 24 h following exposure to cefotaxime (1× MIC), amikacin (1× MIC), and the two drugs in combination were plotted. While the cefotaxime monotherapy resulted in outgrowth of all the bacterial isolates after 8 h, the cefotaxime-amikacin combination presented absolute bactericidal activity, showing rapid diminishment of the bacterial counts within 2 h without any outgrowth by 24 h.

## DISCUSSION

This study for a total of 636 E. coli UTI cases occurring in a month was undertaken to assess the patients with E. coli, UTI-causative E. coli isolates, and the risk factors associated with BSIs secondary to UTI.

E. coli is the most common UTI-causing agent (2), and according to the Kor-GLASS reports, E. coli was found to be the most frequent bacterial species recovered not only from the urine samples but also from the blood samples (12). Increasing rates of cephalosporin resistance in E. coli are of particular concern, and the rate of cefotaxime resistance, 37.6% of E. coli urine isolates in this study, were comparable to the 36.0% of E. coli blood isolates (13). The primary dominant clone ST131 was also the most prevalent clone in E. coli blood isolates, making up a quarter of the total isolates (14). One interesting discrepancy was the composition of the sublineages belonging to ST131. Among E. coli urine isolates, the sublineage H41 was the third most isolated, accounting for more than a quarter of the ST131 isolates, and the sublineage H30 was very rare. However, among the E. coli ST131 blood isolates, three sublineages, H30Rx, H30R, and H30, accounted for approximately 93% of the ST131 isolates and H41 was scarce (14). Curiously, half of the E. coli ST131 H41 isolates possessed the *bla*_CTX-M-14_-like gene. The sublineage H41 was isolated in equal numbers from the eight hospitals, and the proportion of the isolates carrying the *bla*_CTX-M-14_-like gene was even as well. Since the ST131 sublineage H41 has never been reported as a major clone elsewhere in the world and has never been a notorious MDR clone carrying the *bla*_CTX-M_ ESBL gene, the unusual dominance of the E. coli ST131 H41 urine isolates was likely associated with the *bla*_CTX-M-14_-like gene-carrying isolates having a big advantage in clinical settings. The latter dominant clones ST95, ST75, and ST69 were well-known UPEC clones (15).

Among UPEC-related virulence factors, the adhesion- and toxin-associated virulence factors were the focus of screening, since those were likely much associated with the BSIs secondary to UTI (2). For a similar reason, hemolysis activity and the ability to form biofilms were evaluated (2). Notably, the *papC* gene for P fimbriae, which are associated with adhesion to cells (16), was a marked risk factor for the secondary BSIs and frequently found in E. coli ST73. Since E. coli ST73 included fewer drug-resistant isolates, it was likely that the clone was strengthened not by antimicrobial resistance but by virulence to survive in clinical settings.

Cephalosporins are used only in combination with beta-lactamase inhibitors to treat patients with UTIs by ESBL-producing *Enterobacterales* (17). However, amikacin monotherapy is often used to treat patients with UTIs caused by ESBL-producing *Enterobacterales* since the urine isolates are rarely resistant to the drug (18, 19). Amikacin is known to have a bactericidal effect through its targeting of the bacterial ribosome and inhibition of translation by causing misreading and hindering translocation (20), but as demonstrated here through time-kill assay, amikacin monotherapy can result in outgrowth of the bacteria by 24 h. Moreover, for amikacin in combination with cefotaxime, which is hydrolyzed by any type of CTX-M ESBL, a synergistic bactericidal activity was observed in the E. coli isolates carrying either the *bla*_CTX-M-27_ or the *bla*_CTX-M-15_ genes conferring resistance not only to cefotaxime but also to ceftazidime. Of note, the *bla*_CTX-M-14_ gene conferring resistance only to cefotaxime, not to ceftazidime and cefepime, leaves a remaining treatment option.

To the best of our knowledge, this is the first report of the synergistic activities of the cefotaxime-amikacin combination differing by the CTX-M ESBL subtype, and it could be a good option for antimicrobial treatment for patients with E. coli UTI, whose cefotaxime resistance rate is nearly 40%.

This study has several limitations. First, we analyzed E. coli isolates collected in June of one year. Within a month, more than 600 UTI cases were accumulated from eight hospitals, and because of the fully labor-dependent process, further increasing the sampling period was not possible. Second, analysis of paired blood isolates was missing. Additional assessments, such as pulsed-field gel electrophoresis (PFGE) and whole-genome comparison, are left for a further study. Finally, the antimicrobial synergistic effect was evaluated for only three isolates. Even though the isolates were representative dominant strains having characterized resistance determinants, further evaluation for more clinical isolates is needed.

In conclusion, an unexpected widespread UTI-associated bacterial clone, E. coli ST131 H41, was identified with approximately 50% of the sublineage producing group 1 CTX-M ESBL, requiring particular attention. Furthermore, the P fimbria gene, *papC*, which was prevalent in the widespread UPEC clones ST95, ST73, and ST69, was identified as a risk factor for BSIs secondary to UTIs. Even though few dominant STs were identified, no specific STs were associated with bloodstream invasion, indicating that patient risk factors are likely more important in the development of secondary BSIs.

Finally, to the best of our knowledge, we demonstrate for the first time that the effect of drug combination could be differentiated by resistance mechanisms, and we propose the cefotaxime-amikacin combination as a therapeutic alternative for patients with UTIs caused by ESBL-producing E. coli.

## MATERIALS AND METHODS

### Ethics.

The clinical data of the patients including sex, age, type of admission, type of infection, clinical history, comorbidity, clinical symptoms, and lab data, i.e., white blood cell counts, platelet counts, and levels of hemoglobin, bilirubin, and C-reactive protein, were investigated by retrospective chart review devoid of personally identifiable information. Due to the purely observational nature of the study and the very low risk to individual privacy of the participants, this study was approved by all local institutional review boards of the eight sentinel hospitals and exempted from the requirement of informed consent.

### Bacterial isolates.

A total of 636 E. coli isolates, which were obtained from all the UTI cases from eight sentinel hospitals occurring in the month of June 2019, was obtained through the Korea Global Antimicrobial Resistance Surveillance System (Kor-GLASS) (21). All the bacterial isolates were collected through semiquantitative culture of the urine specimens at ≥10^4^ CFU/mL of E. coli in homogeneous growth or at ≥10^5^ CFU/mL of E. coli in heterogeneous growth. Bacterial species were verified using matrix-assisted laser desorption ionization–time of flight mass spectrometry (MALDI-TOF MS) with the Bruker Biotyper (Bruker Daltonics GmbH, Bremen, Germany). The origin of the infections was determined by the calendar days of the patient’s hospitalization, including the previous health care facility before transfer, at the day of urine specimen sampling: hospital origin for ≥2 days and community origin (CO) for <2 days. The BSI secondary to UTI was determined by a subsequent blood culture positive for E. coli with an identical antibiogram.

### Antimicrobial susceptibility testing.

Antimicrobial susceptibility for most drugs, such as ampicillin, piperacillin, ampicillin-sulbactam, cefazolin, cefotaxime, ceftazidime, cefepime, cefoxitin, aztreonam, imipenem, meropenem, ertapenem, amikacin, gentamicin, ciprofloxacin, trimethoprim-sulfamethoxazole, and tigecycline, was determined using the disc diffusion method, and that for colistin was determined using broth microdilution using cation-adjusted Mueller-Hinton broth (CAMHB) (BD, Franklin Lakes, NJ, USA). Interpretation of the susceptibility testing results for drugs followed the guidelines of the Clinical and Laboratory Standards Institute (22), except that for tigecycline interpretation followed the guidelines of the European Committee on Antimicrobial Susceptibility Testing (23). E. coli ATCC 25922 and Pseudomonas aeruginosa ATCC 27853 were used as quality control isolates for the testing. The evaluation of susceptibility was done on *in vitro* analysis only.

### DNA manipulation and molecular typing.

Genomic DNA was extracted from E. coli isolates using the Maxwell 16-cell DNA purification kit (Promega, Madison, WI, USA). STs of each strain were determined using multilocus sequence typing (MLST), with the Achtman scheme (24), by allele numbering the seven housekeeping genes, namely, *adk*, *fumC*, *gyrB*, *icd*, *mdh*, *purA*, and *recA*. A minimum spanning tree was constructed using PHYLOViZ 2.0 (25) with the allelic profile of each ST. Subgroups of the ST131 isolates were identified by (i) *fimH* typing, using the database for the single nucleotide polymorphism (SNP)-based numbering system (26), (ii) ciprofloxacin resistance, and (iii) SNP-based x typing (27). For resistance genotyping, the ESBL *bla*_CTX-M_ gene and the plasmid-mediated *ampC* gene primer pairs were used (21). To genotype the virulence, eight pairs of primers each for adhesin genes, i.e., *afaA*, *papC*, *papG*, and *focG*, and for toxin genes, i.e., *cnf1*, *hlyF*, *sat*, and *cdtB* (2), were designed and used to screen for the presence of the gene.

### Virulence phenotyping.

To detect hemolytic activity of each strain, E. coli isolates were inoculated on 5% sheep agar and incubated at 37°C for 24 h (28). The hemolytic reactions around the inoculum were observed on a light box, in comparison with the quality control isolates E. coli ATCC 25922 and Staphylococcus aureus ATCC 25923 with beta-hemolytic activity. Biofilm formation ability was determined using the Congo red agar test (29). Each E. coli isolate was inoculated by stabbing on brain heart infusion agar (BD) supplemented with 5% (wt/vol) sucrose (Sigma-Aldrich), and 0.08% (wt/vol) Congo red (Sigma-Aldrich), and a strain with black colonies appearing after the incubation at 37°C for 24 h was considered to have an ability to form biofilm.

### Resistance phenotyping.

*In vitro* antimicrobial combinations of the three extended-spectrum cephalosporins and amikacin were assessed using checkerboard analysis on the basis of the fractional inhibitory concentration (FIC) index (30). In a 96-well flat-bottom microplate, three different checkerboard designs, that is, cefotaxime plus amikacin, ceftazidime plus amikacin, and cefepime plus amikacin, were prepared. After inoculating 2 × 10^4^ bacterial cells/well, the plates were incubated at 37°C for 24 h and the MICs of each drug alone and in combinations were determined. The FIC index values were determined and were interpreted as follows: ≤0.5, synergy; 0.5 to 4, additivity; and ≥4, antagonism. Time-kill assay was carried out to evaluate the potential synergistic bactericidal effect of amikacin with cefotaxime. Each isolate at a final concentration of 1 × 10^5^ CFU/mL in MH broth was incubated at 37°C and under four conditions ([i] with 1× MIC of cefotaxime, [ii] with 1× MIC of amikacin, [iii] with 1× MIC of cefotaxime and 1× MIC of amikacin in combination, and [iv] without antimicrobials) for 24 h; subsequently, enumeration of the CFU was carried out after 2, 4, 6, 8, and 24 h of culture. Criteria for defining MDR and XDR in E. coli were based on the number of drugs in an antimicrobial category to which the isolate was nonsusceptible, following the work of Magiorakos et al. (31): MDR refers an isolate nonsusceptible to one or more drugs in more than three antimicrobial classes and XDR indicates an isolate nonsusceptible to one or more drugs in all but two or fewer antimicrobial classes.

### Statistical analyses.

R software (version 4.1.2, http://www.R-project.org/) was used for statistical analyses. The difference between the groups was analyzed using Yates continuity-corrected Pearson’s chi-square test and Fisher’s exact tests for count data. The level of significance for all the comparisons was set at a *P* value of <0.05.

### Data availability.

The data sets generated in this study can be found in the paper.

## References

[1] Klein RD, Hultgren SJ. 2020. Urinary tract infections: microbial pathogenesis, host-pathogen interactions and new treatment strategies. Nat Rev Microbiol 18:211–226. doi:10.1038/s41579-020-0324-0. 32071440 PMC7942789

[2] Flores-Mireles AL, Walker JN, Caparon M, Hultgren SJ. 2015. Urinary tract infections: epidemiology, mechanisms of infection and treatment options. Nat Rev Microbiol 13:269–284. doi:10.1038/nrmicro3432. 25853778 PMC4457377

[3] Riley LW. 2014. Pandemic lineages of extraintestinal pathogenic *Escherichia coli*. Clin Microbiol Infect 20:380–390. doi:10.1111/1469-0691.12646. 24766445

[4] Sante L, Aguirre-Jaime A, Miguel MA, Ramos MJ, Pedroso Y, Lecuona M. 2019. Epidemiological study of secondary bloodstream infections: the forgotten issue. J Infect Public Health 12:37–42. doi:10.1016/j.jiph.2018.08.011. 30266540

[5] Shah C, Baral R, Bartaula B, Shrestha LB. 2019. Virulence factors of uropathogenic *Escherichia coli* (UPEC) and correlation with antimicrobial resistance. BMC Microbiol 19:204. doi:10.1186/s12866-019-1587-3. 31477018 PMC6720075

[6] Brochado AR, Telzerow A, Bobonis J, Banzhaf M, Mateus A, Selkrig J, Huth E, Bassler S, Zamarreno Beas J, Zietek M, Ng N, Foerster S, Ezraty B, Py B, Barras F, Savitski MM, Bork P, Gottig S, Typas A. 2018. Species-specific activity of antibacterial drug combinations. Nature 559:259–263. doi:10.1038/s41586-018-0278-9. 29973719 PMC6219701

[7] Horcajada JP, Shaw E, Padilla B, Pintado V, Calbo E, Benito N, Gamallo R, Gozalo M, Rodríguez-Baño J. 2013. Healthcare-associated, community-acquired and hospital-acquired bacteraemic urinary tract infections in hospitalized patients: a prospective multicentre cohort study in the era of antimicrobial resistance. Clin Microbiol Infect 19:962–968. doi:10.1111/1469-0691.12089. 23279375

[8] Daga AP, Koga VL, Soncini JGM, de Matos CM, Perugini MRE, Pelisson M, Kobayashi RKT, Vespero EC. 2019. *Escherichia coli* bloodstream infections in patients at a university hospital: virulence factors and clinical characteristics. Front Cell Infect Microbiol 9:191. doi:10.3389/fcimb.2019.00191. 31245301 PMC6563721

[9] Greene MT, Chang R, Kuhn L, Rogers MA, Chenoweth CE, Shuman E, Saint S. 2012. Predictors of hospital-acquired urinary tract-related bloodstream infection. Infect Control Hosp Epidemiol 33:1001–1007. doi:10.1086/667731. 22961019 PMC3442945

[10] Jerkeman M, Braconier JH. 1992. Bacteremic and non-bacteremic febrile urinary tract infection–a review of 168 hospital-treated patients. Infection 20:143–145. doi:10.1007/BF01704603. 1644489

[11] Zhang J, Lan P, Yi J, Yang C, Gong X, Ge H, Xu X, Liu L, Zhou J, Lv F. 2021. Secondary bloodstream infection in critically ill patients with COVID-19. J Int Med Res 49:3000605211062783. doi:10.1177/03000605211062783. 34898307 PMC8671686

[12] Lee H, Yoon EJ, Kim D, Jeong SH, Won EJ, Shin JH, Kim SH, Shin JH, Shin KS, Kim YA, Uh Y, Yang JW, Kim IH, Park C, Lee KJ. 2018. Antimicrobial resistance of major clinical pathogens in South Korea, May 2016 to April 2017: first one-year report from Kor-GLASS. Euro Surveill 23:1800047. doi:10.2807/1560-7917.ES.2018.23.42.1800047. PMC6199864 30352640

[13] Kim D, Yoon EJ, Hong JS, Choi MH, Kim HS, Kim YR, Kim YA, Uh Y, Shin KS, Shin JH, Park JS, Park KU, Won EJ, Kim SH, Shin JH, Kim JW, Lee S, Jeong SH. 2021. Major bloodstream infection-causing bacterial pathogens and their antimicrobial resistance in South Korea, 2017–2019: phase I report from Kor-GLASS. Front Microbiol 12:799084. doi:10.3389/fmicb.2021.799084. 35069503 PMC8770956

[14] Yoon EJ, Choi MH, Park YS, Lee HS, Kim D, Lee H, Shin KS, Shin JH, Uh Y, Kim YA, Shin JH, Jeong SH. 2018. Impact of host-pathogen-treatment tripartite components on early mortality of patients with *Escherichia coli* bloodstream infection: prospective observational study. EBioMedicine 35:76–86. doi:10.1016/j.ebiom.2018.08.029. 30139627 PMC6161478

[15] Matsukawa M, Igarashi M, Watanabe H, Qin L, Ohnishi M, Terajima J, Iyoda S, Morita-Ishihara T, Tateda K, Ishii Y, Saga T, Aoki K, Bonomo RA. 2019. Epidemiology and genotypic characterisation of dissemination patterns of uropathogenic *Escherichia coli* in a community. Epidemiol Infect 147:e148. doi:10.1017/S0950268819000426. 30869058 PMC6518783

[16] Dadi BR, Abebe T, Zhang L, Mihret A, Abebe W, Amogne W. 2020. Distribution of virulence genes and phylogenetics of uropathogenic *Escherichia coli* among urinary tract infection patients in Addis Ababa, Ethiopia. BMC Infect Dis 20:108. doi:10.1186/s12879-020-4844-z. 32033541 PMC7006406

[17] Stewart AG, Harris PNA, Henderson A, Schembri MA, Paterson DL. 2020. Oral cephalosporin and β-lactamase inhibitor combinations for ESBL-producing Enterobacteriaceae urinary tract infections. J Antimicrob Chemother 75:2384–2393. doi:10.1093/jac/dkaa183. 32443141

[18] Cho SY, Choi SM, Park SH, Lee DG, Choi JH, Yoo JH. 2016. Amikacin therapy for urinary tract infections caused by extended-spectrum β-lactamase-producing *Escherichia coli*. Korean J Intern Med 31:156–161. doi:10.3904/kjim.2016.31.1.156. 26767869 PMC4712420

[19] Ipekci T, Seyman D, Berk H, Celik O. 2014. Clinical and bacteriological efficacy of amikacin in the treatment of lower urinary tract infection caused by extended-spectrum beta-lactamase-producing *Escherichia coli* or *Klebsiella pneumoniae*. J Infect Chemother 20:762–767. doi:10.1016/j.jiac.2014.08.007. 25179392

[20] Ramirez MS, Tolmasky ME. 2017. Amikacin: uses, resistance, and prospects for inhibition. Molecules 22:2267. doi:10.3390/molecules22122267. PMC5889950 29257114

[21] Lee H, Yoon EJ, Kim D, Jeong SH, Shin JH, Shin JH, Shin KS, Kim YA, Uh Y, Park C, Lee KJ. 2018. Establishment of the South Korean national antimicrobial resistance surveillance system, Kor-GLASS, in 2016. Euro Surveill 23:1700734. doi:10.2807/1560-7917.ES.2018.23.42.1700734. PMC6199867 30352643

[22] CLSI. 2019. Performance standards for antimicrobial susceptibility testing. Twenty-nineth informational supplement N100-S29. CLSI, Wayne, PA.

[23] EUCAST. 2019. Breakpoint tables for interpretation of MICs and zone diameters. Version 9.0, 2019 ed. The European Committee on Antimicrobial Susceptibility Testing.

[24] Wirth T, Falush D, Lan R, Colles F, Mensa P, Wieler LH, Karch H, Reeves PR, Maiden MC, Ochman H, Achtman M. 2006. Sex and virulence in *Escherichia coli*: an evolutionary perspective. Mol Microbiol 60:1136–1151. doi:10.1111/j.1365-2958.2006.05172.x. 16689791 PMC1557465

[25] Nascimento M, Sousa A, Ramirez M, Francisco AP, Carrico JA, Vaz C. 2017. PHYLOViZ 2.0: providing scalable data integration and visualization for multiple phylogenetic inference methods. Bioinformatics 33:128–129. doi:10.1093/bioinformatics/btw582. 27605102

[26] Dias RC, Moreira BM, Riley LW. 2010. Use of *fimH* single-nucleotide polymorphisms for strain typing of clinical isolates of *Escherichia coli* for epidemiologic investigation. J Clin Microbiol 48:483–488. doi:10.1128/JCM.01858-09. 20018817 PMC2815601

[27] Price LB, Johnson JR, Aziz M, Clabots C, Johnston B, Tchesnokova V, Nordstrom L, Billig M, Chattopadhyay S, Stegger M, Andersen PS, Pearson T, Riddell K, Rogers P, Scholes D, Kahl B, Keim P, Sokurenko EV. 2013. Epidemic clonal expansion of CTX-M-15-producing *Escherichia coli* ST131. mBio 4:e00377-13. doi:10.1128/mBio.00377-13. 24345742 PMC3870262

[28] Mogrovejo DC, Perini L, Gostinčar C, Sepčić K, Turk M, Ambrožič-Avguštin J, Brill FHH, Gunde-Cimerman N. 2020. Prevalence of antimicrobial resistance and hemolytic phenotypes in culturable arctic bacteria. Front Microbiol 11:570. doi:10.3389/fmicb.2020.00570. 32318045 PMC7147505

[29] Freeman DJ, Falkiner FR, Keane CT. 1989. New method for detecting slime production by coagulase negative staphylococci. J Clin Pathol 42:872–874. doi:10.1136/jcp.42.8.872. 2475530 PMC1142068

[30] Pillai SK, Moellering RC, Jr, Eliopoulos GM. 2005. Antimicrobial combinations, p 365–440. *In* Lorian V (ed), Antibiotics in laboratory medicine, 5th ed. Lippincott Williams and Wilkins, Philadelphia, PA.

[31] Magiorakos AP, Srinivasan A, Carey RB, Carmeli Y, Falagas ME, Giske CG, Harbarth S, Hindler JF, Kahlmeter G, Olsson-Liljequist B, Paterson DL, Rice LB, Stelling J, Struelens MJ, Vatopoulos A, Weber JT, Monnet DL. 2012. Multidrug-resistant, extensively drug-resistant and pandrug-resistant bacteria: an international expert proposal for interim standard definitions for acquired resistance. Clin Microbiol Infect 18:268–281. doi:10.1111/j.1469-0691.2011.03570.x. 21793988

